# Antimicrobial Resistance in Bacterial Pathogens and Detection of Carbapenemases in *Klebsiella pneumoniae* Isolates from Hospital Wastewater

**DOI:** 10.3390/antibiotics8030085

**Published:** 2019-06-27

**Authors:** Hercules Sakkas, Petros Bozidis, Afrodite Ilia, George Mpekoulis, Chrissanthy Papadopoulou

**Affiliations:** Microbiology Department, Faculty of Medicine, School of Health Sciences, University of Ioannina, 45110 Ioannina, Greece

**Keywords:** Multidrug-resistance, wastewater, carbapenemases, MRSA, VRSA, VRE, ESBL, CKP, immunochromatographic test, PCR

## Abstract

During a six-month period (October 2017–March 2018), the prevalence and susceptibility of important pathogenic bacteria isolated from 12 hospital raw sewage samples in North Western Greece was investigated. The samples were analyzed for methicillin-resistant *Staphylococcus aureus* (MRSA), vancomycin-resistant *enterococci* (VRE), extended-spectrum beta-lactamase (ESBL) producing *Escherichia coli,* carbapenemase-producing *Klebsiella pneumoniae* (CKP), and multidrug-resistant *Pseudomonas aeruginosa.* Antimicrobial susceptibility testing was performed using the agar diffusion method according to the recommendations of the Clinical and Laboratory Standards Institute. The diversity of carbapenemases harboring *K. pneumoniae* was examined by two phenotyping screening methods (modified Hodge test and combined disk test), a new immunochromatographic rapid assay (RESIST-4 O.K.N.V.) and a polymerase chain reaction (PCR). The results demonstrated the prevalence of MRSA, vancomycin-resistant *Staphylococcus aureus* (VRSA), VRE, and CKP in the examined hospital raw sewage samples. In addition, the aforementioned methods which are currently used in clinical laboratories for the rapid identification and detection of resistant bacteria and genes, performed sufficiently to provide reliable results in terms of accuracy and efficiency.

## 1. Introduction

The use of antimicrobials for the treatment of human infections started in the 1900s with Salvarsan (1909) for treatment of syphilis and the discovery of Prontosil (1935), a sulfonamide. Prontosil is the oldest antimicrobial on market and the first to be linked with development of resistance (sulfa drug resistance) due to its extensive use since, Prontosil was the first and only effective antibiotic available in the years before penicillin (1942) [1]. Like sulfonamides, penicillin was extensively used, resulting in the development of resistant bacteria. Between the 1950s and 1970s novel antibiotic classes were discovered and used for the treatment or prevention of human and animal infections. However, the lessons learned from the extensive use of sulfa-drugs and penicillin were not enough to stop the emergence of resistance to new antibiotic classes. As the mortality rates due to multi-drug resistant bacterial infections increase there are concerns that the “golden era of antibiotics” may be followed by the “era with no antibiotics”. The anti-microbial resistance (AMR) phenomenon is currently one of the most important public health issues, and antibiotic resistance genes (ARGs) are considered the emerging pollutant of the environment [2].

ESKAPE bacteria (Enterococcus faecium, Staphylococcus aureus, Klebsiella pneumoniae, Acinetobacter baumannii, Pseudomonas aeruginosa, Enterobacter species) are globally the leading cause of severe healthcare-acquired infections in hospital settings with limited or no antimicrobial treatment options due to AMR [3]. Infected and colonized hospital patients can disseminate ESKAPE bacteria or other drug-resistant bacteria through their excreta usually together with active antimicrobial compounds; thus, hospital effluents constitute the ideal pool for the exchange of resistance genes between clinical and environmental bacteria [4,5]. Studies on the efficiency of wastewater treatment plants show that neither primary or secondary treatment can eliminate efficiently multidrug-resistant bacteria from sewage. Resistant bacteria and genes, surviving the hospital sewage treatment processes can spread and persist in the environment posing a steady reservoir of AMR and a constant health risk for humans and animals [6,7]. 

*Enterococcus* spp. exhibit intrinsic and acquired resistance to various antibiotics, particularly to vancomycin [8]. Vancomycin-resistant enterococci (VRE) capable of spreading resistance genes (*vanA, vanB*) to vancomycin-susceptible strains have emerged worldwide, with several EU countries reporting VRE rates >10% [9] mainly by *E. faecium* [10]. Methicillin-resistant *S. aureus* (MRSA) is reported to have a declining trend in recent years (from 19.6% in 2014 to 16.9% in 2017) in a third of EU countries, but large differences ranging from 1% (Norway) to 44.4% (Romania) are observed among the reporting countries [11]. *A. baumannii* and *P. aeruginosa* are increasingly implicated in hospital-acquired infections exhibiting resistance to most of the available antimicrobial treatments [12]. *Enterobacter* spp. and *E. coli* have the potential to develop mechanisms of antibiotic resistance, including ESBLs and carbapenemases [13,14]. Over the past years, *K. pneumoniae* is considered a major opportunistic pathogen, colonizing the skin, gastrointestinal and respiratory tract, causing a range of severe nosocomial and community-acquired infections like pneumonia and meningitis, as well as wound, urinary tract and bloodstream infections, particularly in neonates, elderly and immunocompromised individuals [15,16]. In patients with *Klebsiella* spp infections, bacteria can be transmitted usually via direct personal contact, medical equipment and contaminated environments [17]. In hospital settings *K. pneumoniae* has been considered one of the most frequent agents of infectious diseases and significant threat to public health because of the high rates of antimicrobial resistance. There are limited data on environmental *Klebsiella* spp isolates, but the existing studies show similarities in phenotypic and genetic characteristics [15,17]. The increasing prevalence of carbapenems-resistant *K. pneumoniae* is a public health concern of major importance in Europe, particularly in Greece reporting the highest percentages (60.5%) of carbapenem-resistant *K. pneumoniae* isolates [18]. The most important beta lactamases conferring resistance to carbapenems that have already been detected in *Enterobacteriaceae* are *K. pneumoniae* carbapenemases (KPC, Ambler class A), metallo-β-lactamases (MBL, Ambler class B) and members of class D β-lactamases such as OXA-48 [19]. 

The aim of the present study was to assess the occurrence of clinically important multidrug-resistant bacteria, including MRSA, vancomycin-resistant *S. aureus* (VRSA), VRE, ESBL-producing bacteria, carbapenemase-producing *K. pneumoniae* (CKP) and *P. aeruginosa,* in raw hospital sewage in order to determine the antimicrobial resistance profile of the isolates, and to define the diversity of carbapenemases harboring *K. pneumoniae* isolates, using a new immunochromatographic rapid assay, two phenotyping screening methods and a ‘gold standard’ molecular method.

## 2. Materials and Methods

### 2.1. Sample Collection and Bacterial Isolation

During a six-month period (October 2017–March 2018), 12 raw untreated sewage samples from a tertiary hospital (Ioannina, Greece) were collected. Samples of 0.5 L each were placed into sterile bottles, transported to the laboratory on ice packs in portable insulated containers and processed within 24 h. Samples were concentrated using the filtration technique onto 0.45-μm pore size filter membranes (Merck, Darmstadt, Germany). Membranes were placed onto the surface of differential selective agars; MacConkey agar supplemented with 1μg/ml imipenem (Oxoid Ltd., Basingstoke, UK), Chapman and Bile Aesculin media, (Oxoid Ltd., Basingstoke, UK). Plates were incubated for 24 h at 37 °C in air. Selected colonies were transferred to selective chromogenic media CHROMagar MRSA, CHROMagar VRE, CHROMagar ESBL, CHROMagar KPC, CHROMagar Pseudomonas (CHROMagar, Paris, France) (24 h, 37 °C). The identification of isolated species was determined using the API Staph, API 20 STREP and API 20E identification systems (bioMerieux SA, Marcy-l’Etoile, France).

### 2.2. Antimicrobial Susceptibility Testing

Antimicrobial susceptibility testing was performed by applying the agar diffusion method according to the Clinical and Laboratory Standards Institute (CLSI) recommendations [20], including the following antibiotics: Penicillin G (10 U), ampicillin (10 μg), amoxicillin/clavulanate (20/10 μg), piperacillin/tazobactam (100/10 μg), ticarcillin/clavulanate (75/10 μg), vancomycin (30 μg), teicoplanin (30 μg), erythromycin (15 μg), clindamycin (2 μg), amikacin (30 μg), gentamicin (10 μg), tobramycin (10 μg), cefoxitin (30 μg), ceftriaxone (30 μg), ceftazidime (30 μg), cefepime (30 μg), ciprofloxacin (5 μg), imipenem (10 μg), meropenem (10 μg), quinupristin/dalfopristin (15 μg), linezolid (30 μg), colistin (10 μg), aztreonam (30 μg), trimethoprim/sulfamethoxazole (1.25/23.75 μg), tetracycline (30 μg) (Oxoid Ltd., Basingstoke, UK). *S. aureus* strains were tested against penicillin G, erythromycin, clindamycin, gentamicin, cefoxitin, ciprofloxacin, quinupristin/dalfopristin, whereas *Enterococcus* species were examined against penicillin G, ampicillin, vancomycin, teicoplanin, erythromycin, ciprofloxacin, quinupristin/dalfopristin, linezolid and tetracycline. Antimicrobial susceptibility testing for Enterobacteriaceae included amoxicillin/clavulanate, amikacin, gentamicin, cefoxitin, ceftriaxone, ceftazidime, cefepime, ciprofloxacin, aztreonam, trimethoprim/sulfamethoxazole, tetracycline, ampicillin (for *E. coli*) and tobramycin (for *Klebsiella* spp). *P. aeruginosa* isolates were tested against piperacillin/tazobactam, ticarcillin/clavulanate, amikacin, gentamicin, tobramycin, ceftazidime, cefepime, ciprofloxacin, imipenem, meropenem and aztreonam. In addition, the minimum inhibitory concentration (MIC) test was performed to determine the resistance of MRSA to vancomycin and teicoplanin, the resistance of *K. pneumoniae* to imipenem, meropenem, and tigecycline, as well as the resistance of *P. aeruginosa* to colistin (Liofilchem, Roseto degli Abruzzi (TE), Italy) following the CLSI breakpoints [20]. 

### 2.3. Phenotyping Screening and Carbapenemases Detection

Imipenem and meropenem resistant *Klebsiella* isolates were tested for carbapenemases production using: (1) the modified Hodge test (MHT), (2) the meropenem combined disk test (CDT), (3) the RESIST-4 O.K.N.V immunochromatographic test (IT) and (4) a multiplex PCR assay. 

MHT is a relatively simple screening test for routine detection of carbapenemases and it is based on the inactivation of a carbapenem by carbapenemase-producing strains that enable a carbapenem-susceptible indicator strain (*E. coli* ATCC 25922 strain), to extend growth towards a carbapenem-containing disc along the streak of inoculum of the test strain. A positive test result gives a clover leaf-like indentation. The MHT was performed according to the following procedure: a 1:10 dilution of 0.5 McFarland *E. coli* ATCC 25922 inoculum was placed onto Mueller Hinton agar (MHA) (Oxoid Ltd., Basingstoke, UK) surface with a disk of meropenem (10 μg) in the center of the plate. *K. pneumoniae* ATCC BAA-1705 was used as a positive control. The *Klebsiella* isolates to be tested were inoculated, each one in a straight line from the edge of the meropenem disk to the edge of the plate; they were incubated overnight in ambient air (37 °C, 24 h) and an interpretation of the results followed. The result was considered positive (indicating carbapenemase production by the test isolate) when a clover leaf-type indentation within the zone of inhibition of the meropenem susceptibility disk [20]. 

CDT is routinely used for differentiating carbapenemase-producing enterobacteria, and in this study was performed as previously described by Pournaras et al. [21]; MHA was inoculated with 0.5 McFarland dilution of each carbapenem-resistant strain, and disks of meropenem (10 μg) alone, meropenem with phenylboronic acid (PBA), meropenem with EDTA and meropenem with both PBA and EDTA (Liofilchem, Roseto degli Abruzzi (TE), Italy) were placed onto the surface of plates [22]. Three CKP from our collection (KPC, VIM and NDM-producing *K. pneumoniae* were used as positive controls). After incubation (37 °C, 24 h), a 5-mm difference between the inhibition zones of meropenem disk with the inhibitors PBA, EDTA and PBA/EDTA and meropenem alone, was considered as a positive result for detection of KPC, MBL or KPC/MBL carbapenemases respectively [21].

RESIST-4 O.K.N.V (CORIS, BioConcept, Gembloux, Belgium) can detect the four most prevalent carbapenemases (OXA-48-like, KPC, NDM, VIM). Detection of CKP isolates by RESIST-4 O.K.N.V was performed on pure cultures grown on MacConkey agar for 24 h, according to the manufacturer’s recommendations. This recently developed rapid test [23,24,25,26,27,28] is based on membrane technology with colloidal gold nanoparticles. Each test consists of two lateral-flow cassettes with a nitrocellulose membrane which is sensitized with monoclonal antibodies against KPC, OXA-48, NDM and VIM carbapenemases. After the preparation procedure and a 15-minute reaction, the appearance of a reddish-purple band at test line positions confirms a positive result. Quality control was performed by using the three aforementioned positive CDT clinical isolates and an OXA-48-like-producing clinical isolate from our collection.

A previously published multiplex PCR with primers targeting the *bla*_VIM_, *bla*_OXA-48_, *bla*_NDM_, and *bla*_KPC_ genes [29] was used. The final volume of 50 μL PCR reaction was containing 2–3 μL of total DNA, 1× HotStarTaq PCR buffer (Tris pH 8.7, KCl, (NH_4_)_2_SO_4_, 1.5mM MgCl_2_, 200 μM dNTP, 40pmol of each of the VIM-F, VIM-R, OXA-F, OXA-R, NDM-F, NDM-R, KPC-F and KPC-R specific primers [29] and 1U of HotStarTaq DNA polymerase (Qiagen, Germantown, USA). The amplification program involved an initial denaturation at 94 °C for 10 min followed by 36 cycles of 94 °C for 30 s, 52 °C for 40 s at 72 °C for 50 s with a final extension at 72 °C for 50 s. The same program was used for the identification of Enterococcal species *E. faecalis* and *E. faecium* using the specific primers DD13, DD3-2 and FAC1-1, FAC2-1 accordingly [30]. All PCR products were analyzed by electrophoresis on a 2% agarose gel in 1× TBE buffer stained with ethidium bromide (10 μM) and visualized under UV light.

## 3. Results

In total, 70 resistant isolates (13 Gram positive and 57 Gram negative) were identified; *S. aureus* (*n* = 6), *Enterococcus* spp (*n* = 7), *E. coli* (*n* = 24), *K. pneumoniae* (*n* = 24) and *P. aeruginosa* (*n* = 9). All *S. aureus* isolates were resistant to both penicillin and oxacillin (tested by cefoxitin disk test). Among the six MRSA strains one was also resistant to vancomycin (16.7%), while the overall prevalence of VRE was 57.1%. In addition, nine (100%), 23 (95.8%) and 20 (83.3%) of all *P. aeruginosa*, *K. pneumoniae* and *E. coli* isolates respectively, were identified as multidrug-resistant (resistance to three or more antibiotics classes tested), whereas five isolates out of 57 Gram negative bacteria (*E. coli*, *n* = 4 and *K. pneumoniae*, *n* = 1) were not classified as multidrug-resistant (8.8%). Colistin revealed the lowest resistance in *P. aeruginosa* isolates (*n* = 2, 22.2%) and 19 out of 24 *K. pneumoniae* isolates were resistant to carbapenems (79.2%), while one out of the 19 carbapenem- resistant isolates was resistant to tigecycline (5.3%) (Table 1, Tables S1–S5).

**Figure 1:**
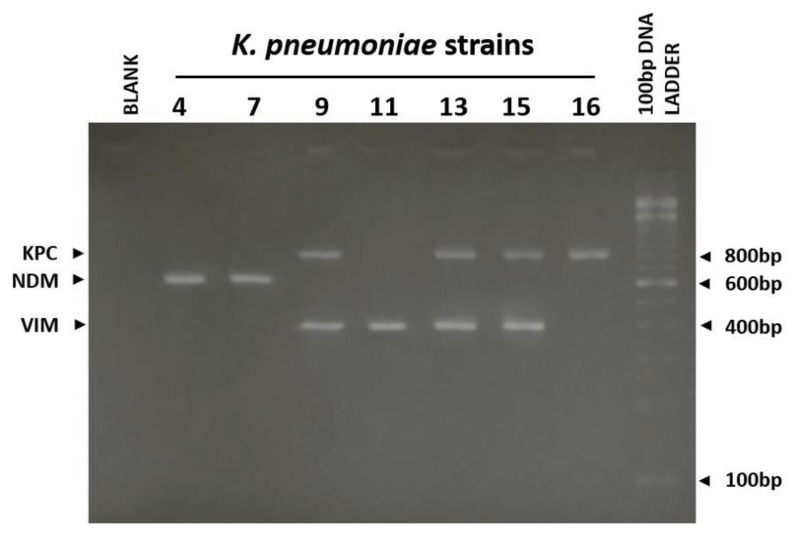
Agarose gel electrophoresis (2% *w/v*) used for the separation of PCR products produced by a multiplex PCR reaction against *bla*_KPC_, *bla*_NDM*,*_ *bla*_OXA-48_ and *bla*_VIM_ genes. Blank corresponds to a negative control (without DNA). Bands correspond to PCR products from PCR screening of *K.pneumoniae* isolates 4,7,9,11,13,15 and isolate 16. The size of each amplicon is indicated on the left. The ladder used is the 100 bp DNA ladder (InvitrogenTM, ThermoFisher Scientific).

The molecular screening of carbanemase genes was based on a previously published multiplex PCR technique [29]. Nineteen *K. pneumoniae* isolates positive for class A (*bla*_KPC_) and class B (*bla*_NDM_ and *bla*_VIM_) genes by immunochromatographic test (IT) were screened against *bla*_KPC,_ *bla*_NDM,_ *bla*_OXA-48_ and *bla*_VIM_ genes using the appropriate primers. The multiplex PCR reaction produced amplicons with the expected length of 798bp, 621bp, 438bp and 390bp respectively (Figure 1). RESIST-4 O.K.N.V and PCR assays identified the same carbapenemase gene in 17 out of the 19 (89.5%) isolates. The immunoassay detected the *bla*_KPC_ or *bla*_VIM_ genes in two samples (10.5%), which were positive for both genes by PCR. Νone of these isolates was positive for *bla*_OXA-48_ carbapenemase gene by both methods. 

Regarding the phenotypic screening tests, MHT revealed the production of carbapenemases in 15 out of 19 *K. pneumoniae* isolates (78.9%), whereas the CDT identified all the carbapenemase-producing *K. pneumoniae* isolates. However, in three out of 19 isolates (18.7%), CDT was considered positive for detection of both KPC and MBL carbapenemases, while only MBL enzymes were detected by PCR. The results from PCR, MHT, CDT and IT tests are presented in Table 2.

A second PCR reaction was used for the identification of the isolated enterococci strains. The primers could amplify specific ddl genes producing amplicons of 475 bp or 1091 bp length for *E. faecalis* or *E. faecium* correspondingly. All seven *Enterococcus* spp isolates were identified as *E. faecalis.*

## 4. Discussion

AMR is becoming the most significant public health problem of the 21st century and is linked to factors related to the overuse or misuse of antimicrobials in human and veterinary practice, and to environmental pollution. Addressing the AMR phenomenon effectively requires close collaboration under a “one health” approach, taking into account the interconnections between human health, animal health and the environment. Most often, AMR occurs in healthcare settings due to non-prudent use of antimicrobials and the lack of effective infection control policy. The role of the aquatic environment as a route of pathogens transmission has long been recognized and it is commonly associated with effluents of wastewaters from hospitals, animal breeding farms and urban sewage infrastructures. Yet, there is no robust evidence on the circumstances or pathways initiating the emergence and transmission of drug-resistant strains to humans [31,32]. Recent studies show that not only bacteria but also bacterial genes can move freely among humans, animals and the environment [33,34,35]. Hence the risk of AMR transmission from the environment to humans has to be assessed based on both the resistant bacteria and the resistance genes circulating in the environment. In our study we examined both resistant bacteria and specific resistance genes focusing on clinically important strains in order to contribute more data in this field. 

There are limited published data relating to the dissemination of clinically important MRSA, VRSA and VRE isolates and resistance genes from hospital and urban wastewater treatment plants and other sources such as farm animals and foods of animal origin [36,37,38,39,40,41]. The issue of resistant staphylococci and enterococci is of outmost importance, because both microorganisms have emerged as predominant pathogens in hospital infections during the last two decades. Ιn the present study, the rate of MRSA strains demonstrating resistance against fluoroquinolones and macrolides was very high (100%), while the prevalence of VRSA strains was rather low (16.7%). Mandal et al. [38] reported 90% MRSA and 15% VRSA from hospital effluents in West Bengal, while Gomez et al. [40] reported only one isolation of MRSA strain from urban wastewater treatment plants in La Rioja, Spain. Two studies at Thibodaux (Louisiana, USA) showed persistent presence of MRSA and resistance genes in both raw and treated sewage [39,41]. Little information is available in relation to the prevalence of VRE strains in non-hospital environments. A publication relating to VRE and VanA genes in the aquatic environment has been reported in Pinellas County (Florida, USA) after an accidental release of untreated sewage [42]. In another study, VRE with similar multidrug-resistant patterns were isolated from patients, hospital effluents and urban wastewater, providing strong evidence of spreading such resistant bacteria from hospital sewage to the environment [43].

In our study the resistant Gram-negative rods (GNR) were a common finding, confirming their increased prevalence in hospital-associated drug-resistant infections. The reported rise of ESBL and carbapenemase-producing Enterobacteriaceae and non-fermenting GNR, mainly *E. coli*, *K. pneumoniae, Enterobacter* spp, *A. baumannii* and *P. aeruginosa* in hospital wastewater treatment plants is a matter of particular health concern but it has gained research attention only recently [7,44,45,46,47,48,49]. There are few studies assessing the presence of resistant GNR and resistant genes in hospital wastewaters while data on their occurrence in untreated hospital wastewaters are scarcer. Taking into account that during the last fifteen years the aforementioned GNR have become of major health concern as principal agents of drug-resistant infections, any evidence on their presence in untreated hospital wastewater is important. In our study, the commonest resistant isolates correspond to *E. coli* and *K. pneumoniae.* In particular for *K. pneumoniae*, we found that most isolates (79.2%) encoded class A (*bla*_KPC_) carbapenemase—which is currently the most prevalent—and class B (*bla*_NDM_ and *bla*_VIM_) enzymes, while co-production of both KPC and MBL genes (Table 2), was demonstrated in eight strains (42.1%); this observation coincides with the findings reported for clinical isolates by other researchers in Greece [22,50].

Interestingly, the majority (77.8%) of the multiresistant *P. aeruginosa* strains were susceptible to colistin (Table 1), a polymyxin which has been used extensively in the past (1940s–1970s) against Gram-negative bacteria but was abandoned because of its nephrotoxic and neurotoxic side-effects. However, this forgotten drug got back in use in the early 2000s due to the emergence of carbapenem-resistant Gram-negative bacteria which were found to be susceptible to polymyxins [51]. Unfortunately, as the use of colistin increased, the colistin resistance among carbapenem-resistant GNR increased as well [52]. Two recent multicenter clinical and laboratory studies on carbapenem-resistant *K. pneumoniae* isolates from medical centers in U.S.A revealed colistin-resistance in 13% and 16% of the isolates [53,54]. In the present study colistin-resistance was observed in 22.2% of the *P. aeruginosa* isolates (Table 1) indicating colistin-resistance development in this country too. Yet, since the prospect of novel alternative antimicrobials for treatment of multidrug-resistant infections in the near future is uncertain, colistin can act as an effective antipseudomonal agent. In our study one carbapenem-resistant *K. pneumoniae* isolate was found resistant to tigecycline (5.3%), a glycylcycline antibiotic which is currently used as a last-line treatment in severe infections caused by multidrug-resistant Gram-positive and Gram-negative pathogens. In two previous studies tigecycline was the most appropriate therapeutic approach against infections caused by carbapenem-resistant *K. pneumoniae,* among intensive care unit and tertiary care center patients [55,56]. It has been reported that the combination treatment scheme of both colistin and tigecycline may act synergistically against carbapenemem-resistant infections providing low rates of mortality [57].

Tracking the transportation of carbapenemase resistance genes among hospital settings, treated and untreated wastewaters, and the environment is important for planning effective surveillance, prevention and infection control strategies but requires methods easily applied in routine labs. An accurate and cost-effective assessment of carbapenemases’ production is of urgent need, particularly in high-endemic regions. In our study we used a new rapid immunochromatographic assay and two standard phenotyping methods (MHT, CDT) to determine the type of produced carbapenemases. Results obtained by the aforementioned techniques were compared to those from a multiplex PCR assay which is recognized as the “gold standard” method. The employed phenotypic methods are considered useful, rapid, and cost-effective for the detection of carbapenemases; however, according to our results (Table 2), although rapid and inexpensive, they do not provide specific information about the type of carbapenemases. CDT detects KPC and MBL carbapenemases and thus is more sensitive than MHT which detects carbapenemases without differentiating them. In previous studies CDT exhibited sensitivity of 94.8% (87.8%–98%) and specificity of 100% (95–100%) for detecting carbapenemase-producing Enterobacteriaceae [21], while MHT’s sensitivity and specificity were 92.7% and 63.4% respectively [58]. The IT rapid assay performed remarkably well producing reliable results, almost identical with those produced by the PCR. The two methods produced different results for only two out of the 19 *K. pneumoniae* strains examined, with IT detecting one carbapenemase gene instead of two detected by PCR (Table 2, Figure 1). According to our results, and taking into consideration that rapid assays are extensively used in point-of-care testing, saving time and producing quite reliable results, they can be used for specimen other than clinical. The IT used in the present study performed very sufficiently and could be used for the fast detection and characterization of the four most commonly produced *K. pneumoniae* carbapenemases in wastewater samples, especially when PCR is not available in the laboratory.

## 5. Conclusions

In conclusion, our results demonstrated the presence of important clinical pathogens in raw hospital sewage, which are likely to be released in the environment. Hospital wastewaters are considered “hot spots” of antibiotic resistant bacteria, resistance genes, and mobile genetic elements no matter whether they are treated or not, since there are different sewage treatment technologies which may have diverse effects on microbial communities; in this respect there is a knowledge gap regarding the fate of resistant bacteria and genetic elements during wastewater treatment processes. Still there remain several further knowledge gaps that have to be filled in order to control and mitigate efficiently the emergence and spread of AMR in the environment. AMR is developing through complex interactions including de-novo mutation under clinical antibiotic pressure, the acquisition of mobile genes that evolve progressively in environmental bacteria, rapid demographic changes, human and animal population movement, as well as agricultural, landscape, climate and environmental changes. Giving more attention to how anthropogenic activities are influencing the evolution of antimicrobial resistance and broad, multisectoral and interdisciplinary collaboration are key-factors in addressing the rise of AMR.

## Figures and Tables

**Table 1:** Antibiotic resistance (%) profile of tested bacteria.

Bacteria/Antibiotics *	*S.aureus* (*n* = 6)	*Enterococcus* spp (*n* = 7)	*E. coli* (*n* = 24)	*K. pneumoniae* (*n* = 24)	*P. aeruginosa* (*n* = 9)
PEN	100%	57.1%			
AMP		85.7%	100%		
AMC			79.2%	75%	
PTZ					55.5%
TCC					77.7%
VAN	16.7%	57.1%			
TEC	16.7%	57.1%			
ERY	100%	71.4%			
CC	100%				
AMK			25%	62.5%	88.8%
GEN	66.6%		29.2%	45.8%	88.8%
TOB				100%	100%
FOX	100%		66.6%	83.3%	
CRO			100%	100%	
CAZ			70.8%	95.8%	33.3%
FEP			54.2%	83.3%	66.6%
CIP	100%	71.4%	79.2%	87.5%	88.8%
IPM				79.2%	88.8%
MEM				79.2%	100%
ATM			45.8%	58.3%	55.5%
QDA	33.3%	100%			
LZD		85.7%			
SXT			66.6%	95.8%	
CT					22.2%
TET		71.4%	45.8%	58.3%	
TGC				5.3%	

* PEN, penicillin G; AMP, ampicillin; AMC, amoxicillin/clavulanate; PTZ, piperacillin/tazobactam; TCC, ticarcillin/clavulanate; VAN, vancomycin; TEC, teicoplanin; ERY, erythromycin; CC, clindamycin; AMK, amikacin; GEN, gentamicin; TOB, tobramycin; FOX, cefoxitin; CRO, ceftriaxone; CAZ, ceftazidime; FEP, cefepime; CIP, ciprofloxacin; IPM, imipenem; MEM, meropenem; ATM, aztreonam; QDA, quinupristin/dalfopristin; LZD, linezolid; SXT, trimethoprim/sulfamethoxazole; CT, colistin; TET, tetracycline; TGC, tigecycline.

**Table 2:** Phenotyping screening and carbapenemases detection in *K.pneumoniae* strains by immunochromatographic rapid assay and PCR.

*K. pneumoniae*	MHT	CDT (β-lactamase detected)	IT	PCR
KP1	+	KPC/MBL	VIM	VIM
KP2	+	KPC	KPC	KPC
KP3	+	KPC	KPC	KPC
KP4	−	MBL	NDM	NDM
KP5	+	KPC/MBL	KPC+NDM	KPC+NDM
KP6	−	KPC/MBL	KPC+NDM	KPC+NDM
KP7	−	MBL	NDM	NDM
KP8	+	KPC/MBL	KPC+VIM	KPC+VIM
KP9	+	KPC/MBL	KPC+VIM	KPC+VIM
KP10	+	KPC	KPC	KPC
KP11	+	KPC/MBL	VIM	VIM
KP12	+	MBL	VIM	VIM
KP13	+	KPC/MBL	VIM	KPC+VIM
KP14	+	KPC/MBL	KPC+VIM	KPC+VIM
KP15	+	KPC/MBL	KPC	KPC+VIM
KP16	+	KPC	KPC	KPC
KP17	+	KPC/MBL	VIM	VIM
KP18	+	KPC	KPC	KPC
KP19	−	KPC/MBL	KPC+VIM	KPC+VIM

MHT: Modified Hodge test, CDT: combined disk test, IT: immunochromatographic test.

## Supplementary Materials

The following are available online at https://www.mdpi.com/2079-6382/8/3/85/s1, Table S1: Antibiotic resistance profile of *Staphylococcus aureus*, Table S2: Antibiotic resistance profile of *Enterococcus* spp., Table S3: Antibiotic resistance profile of *Escherichia coli*. Table S4: Antibiotic resistance profile of *Klebsiella pneumoniae*. Table S5: Antibiotic resistance profile of *Pseudomonas aeruginosa*.
