# Supplementary material for: Antimicrobial Resistance in Bacterial Pathogens and Detection of Carbapenemases in Klebsiella pneumoniae Isolates from Hospital Wastewater
Source: Antibiotics (Basel). 2019 Jun 27;8(3):85. doi: 10.3390/antibiotics8030085 (PMC6783922; doi:10.3390/antibiotics8030085)
Supplement: Supplementary file 1 [file antibiotics-08-00085-s001.pdf]

Supplementary Files

Table S1. Antibiotic resistance profile of *Staphylococcus aureus*.

Isolate No./Antibiotics	PEN	VAN	TEC	ERY	CIP	CC	QDA	FOX	GEN
1	R	S	S	R	R	R	S	R	S
2	R	S	S	R	R	R	S	R	S
3	R	S	S	R	R	R	S	R	R
4	R	S	S	R	R	R	S	R	R
5	R	S	S	R	R	R	R	R	R
6	R	R	R	R	R	R	R	R	R

PEN, penicillin G; VAN, vancomycin; TEC, teicoplanin; ERY, erythromycin; CIP, ciprofloxacin; CC, clindamycin; QDA, quinupristin/dalfopristin; FOX, cefoxitin; GEN, gentamicin.

S: Susceptible; R: Resistant.

Table S2. Antibiotic resistance profile of *Enterococcus* spp.

Isolate No./Antibiotics	PEN	AMP	VAN	TEC	ERY	CIP	QDA	LZD	TET
1	R	R	R	R	R	R	R	R	R
2	R	R	R	R	R	R	R	R	R
3	S	S	S	S	R	S	R	S	S
4	R	R	R	R	R	R	R	R	R
5	S	R	S	S	S	S	R	R	S
6	S	R	S	S	S	R	R	R	R
7	R	R	R	R	R	R	R	R	R

PEN, penicillin G; AMP, ampicillin; VAN, vancomycin; TEC, teicoplanin; ERY, erythromycin; CIP, ciprofloxacin; QDA, quinupristin/dalfopristin; LZD, linezolid; TET, tetracycline.

S: Susceptible; R: Resistant.

Table S3. Antibiotic resistance profile of *Escherichia coli*.

Isolate No./Antibiotics	AMP	AMC	AMK	GEN	FOX	CRO	CAZ	FEP	CIP	ATM	SXT	TET
1	R	R	S	S	R	R	R	R	R	S	R	S
2	R	R	R	R	R	R	R	R	R	S	R	R
3	R	R	S	S	R	R	R	R	R	R	R	S
4	R	R	S	S	I	R	R	I	R	R	R	R
5	R	R	S	S	R	R	R	R	R	I	R	R
6	R	R	S	S	R	R	R	R	S	R	R	R
7	R	R	S	S	R	R	R	S	R	S	S	R
8	R	R	S	S	R	R	R	R	R	R	S	S
9	R	R	R	I	R	R	R	R	R	R	R	S
10	R	R	R	R	R	R	I	I	R	S	R	R
11	R	S	S	S	R	R	R	R	S	S	S	S
12	R	S	S	S	S	R	S	S	I	S	I	I
13	R	R	S	S	R	R	R	R	R	R	S	S
14	R	R	S	S	S	R	S	S	R	R	R	R

15	R	R	R	R	R	R	R	R	R	I	R	I
16	R	R	R	R	R	R	R	R	R	S	S	R
17	R	R	S	R	I	R	S	S	R	R	R	R
18	R	R	S	S	S	R	S	S	R	R	R	R
19	R	S	S	S	S	R	S	S	R	R	R	R
20	R	R	I	R	I	R	S	I	R	R	R	S
21	R	R	R	R	R	R	R	R	R	S	R	S
22	R	S	S	S	R	R	R	S	S	S	S	S
23	R	S	S	S	I	R	R	S	S	S	S	S
24	R	R	S	S	R	R	R	R	R	S	R	S

AMP, ampicillin; AMC, amoxicillin/clavulanate; AMK, amikacin; GEN, gentamicin; FOX, cefoxitin; CRO, ceftriaxone; CAZ, ceftazidime; FEP, cefepime; CIP, ciprofloxacin; ATM, aztreonam; SXT, trimethoprim/sulfamethoxazole; TET, tetracycline.

S: Susceptible; R: Resistant; I: Intermediate.

Table S4. Antibiotic resistance profile of *Klebsiella pneumoniae.*

Isolate No./Antibiotics	AMC	AMK	GEN	TOB	FOX	CRO	CAZ	FEP	CIP	IMP	MEM	ATM	SXT	TET	TGC
1	R	R	R	R	R	R	R	R	R	R	R	I	R	S	S
2	R	I	S	R	R	R	R	R	R	R	R	R	R	R	S
3	R	R	R	R	R	R	R	R	R	R	R	R	R	R	S
4	R	R	R	R	R	R	R	R	R	R	R	S	R	S	S
5	R	S	I	R	R	R	R	R	R	R	R	R	R	R	S
6	R	R	R	R	R	R	R	R	R	R	R	R	R	R	S
7	R	I	I	R	R	R	R	R	R	R	R	R	R	R	S
8	R	R	R	R	R	R	R	R	R	R	R	R	R	R	S
9	R	R	R	R	R	R	R	R	R	R	R	R	R	S	S
10	R	S	I	R	R	R	R	R	R	R	R	R	R	R	S
11	R	R	I	R	R	R	R	R	R	R	R	S	R	R	S
12	R	R	R	R	R	R	R	R	R	R	R	S	R	R	S
13	R	R	I	R	R	R	R	R	R	R	R	R	R	R	S
14	R	R	R	R	R	R	R	R	R	R	R	R	R	R	R

15	R	R	R	R	R	R	R	R	R	R	R	R	R	S	S
16	R	R	I	R	R	R	R	R	R	R	R	R	R	R	S
17	R	R	R	R	R	R	R	R	R	R	R	S	R	S	S
18	R	S	S	R	R	R	R	R	R	R	R	R	R	R	S
19	I	R	R	R	R	R	R	R	R	R	R	R	R	R	S
20	S	S	S	R	I	R	R	I	R	S	S	S	R	S	NT
21	I	R	I	R	I	R	R	I	S	S	S	S	R	S	NT
22	S	I	I	R	R	R	R	R	S	S	S	S	R	S	NT
23	S	S	S	R	S	R	R	I	R	S	S	S	R	S	NT
24	S	S	S	R	S	R	S	S	S	S	S	S	S	S	NT

AMC, amoxicillin/clavulanate; AMK, amikacin; GEN, gentamicin; TOB, tobramycin; FOX, cefoxitin; CRO, ceftriaxone; CAZ, ceftazidime; FEP, cefepime; CIP, ciprofloxacin; IPM, imipenem; MEM, meropenem; ATM, aztreonam; SXT, trimethoprim/sulfamethoxazole; TET, tetracycline; TGC, tigecycline.

S: Susceptible; R: Resistant; I: Intermediate; NT: Not tested.

Table S5. Antibiotic resistance profile of *Pseudomonas aeruginosa.*

Isolate No./Antibiotics	PTZ	TCC	AMK	GEN	TOB	CAZ	FEP	CIP	IMP	MEM	ATM	CT
1	S	S	S	S	R	S	S	R	R	R	S	S
2	R	R	R	R	R	S	R	R	R	R	R	R
3	S	S	R	R	R	R	R	S	R	R	S	S
4	R	R	R	R	R	S	S	R	R	R	S	S
5	R	R	R	R	R	S	R	R	R	R	R	S
6	S	R	R	R	R	S	S	R	R	R	S	S
7	R	R	R	R	R	S	R	R	R	R	R	S
8	R	R	R	R	R	R	R	R	S	R	R	R
9	S	R	R	R	R	R	R	R	R	R	R	S

PTZ, piperacillin/tazobactam; TCC, ticarcillin/clavulanate; AMK, amikacin; GEN, gentamicin; TOB, tobramycin; CAZ, ceftazidime; FEP, cefepime; CIP, ciprofloxacin; IPM, imipenem; MEM, meropenem; ATM, aztreonam; CT, colistin.

S: Susceptible; R: Resistant.
